# TRAP150 interacts with the RNA-binding domain of PSF and antagonizes splicing of numerous PSF-target genes in T cells

**DOI:** 10.1093/nar/gkv816

**Published:** 2015-10-10

**Authors:** Christopher A. Yarosh, Iulia Tapescu, Matthew G. Thompson, Jinsong Qiu, Michael J. Mallory, Xiang-Dong Fu, Kristen W. Lynch

**Affiliations:** 1 Department of Biochemistry and Biophysics Perelman, School of Medicine, University of Pennsylvania, Philadelphia, PA 19104, USA; 2 Department of Cell and Molecular Medicine, University of California, San Diego, San Diego, CA 92093, USA; 3 Department of Genetics, Perelman School of Medicine, University of Pennsylvania, Philadelphia, PA 19104, USA

## Abstract

PSF (a.k.a. SFPQ) is a ubiquitously expressed, essential nuclear protein with important roles in DNA damage repair and RNA biogenesis. In stimulated T cells, PSF binds to and suppresses the inclusion of CD45 exon 4 in the final mRNA; however, in resting cells, TRAP150 binds PSF and prevents access to the CD45 RNA, though the mechanism for this inhibition has remained unclear. Here, we show that TRAP150 binds a region encompassing the RNA recognition motifs (RRMs) of PSF using a previously uncharacterized, 70 residue region we have termed the PSF-interacting domain (PID). TRAP150's PID directly inhibits the interaction of PSF RRMs with RNA, which is mediated through RRM2. However, interaction of PSF with TRAP150 does not appear to inhibit the dimerization of PSF with other *Drosophila* Behavior, Human Splicing (DBHS) proteins, which is also dependent on RRM2. Finally, we use RASL-Seq to identify ∼40 T cell splicing events sensitive to PSF knockdown, and show that for the majority of these, PSF's effect is antagonized by TRAP150. Together these data suggest a model in which TRAP150 interacts with dimeric PSF to block access of RNA to RRM2, thereby regulating the activity of PSF toward a broad set of splicing events in T cells.

## INTRODUCTION

An emerging theme in the study of gene regulation is the importance of controlling the activity of RNA-binding proteins (RBPs) (1). Human cells express hundreds of RBPs that regulate virtually every aspect of RNA biogenesis and processing, from transcription to translation and decay (2). The differential activity of these proteins thus dictates which messages are expressed and translated in distinct cells or in response to different growth conditions. However, the underlying cellular strategies for controlling these proteins are underexplored, limiting our understanding of how these proteins can steer the many different nuclear events that guarantee cell viability.

One RBP that is regulated in a cell-state dependent manner is PSF, or SFPQ (PTB-associated Splicing Factor/Splicing Factor Proline-Glutamine rich) (3). PSF is a ubiquitously expressed, essential nuclear protein that is a member of the DBHS (Drosophila Behavior Human Splicing) family of proteins, which in vertebrates also includes p54^nrb^/NONO and PSPC1 (3–5). The DBHS proteins all share a core domain block consisting of a tandem pair of RNA-recognition motifs (RRMs), a protein–protein interaction domain known as a NONA/Paraspeckle (NOPS) domain, and a stretch of amino acids known to form coiled-coil interactions in DBHS oligomers (5,6). PSF stands apart from the other DBHS proteins, however, in that it also contains a large low complexity, proline-rich region N-terminal to the core domain, a linker region between the proline-rich sequence and RRMs (PR-linker) and an extended C-terminus that includes two nuclear localization signals and areas of predicted protein flexibility (3). PSF's distinct domain arrangement, together with its broad ability to bind DNA and RNA, enables its participation in a host of nuclear functions ranging from DNA double strand break repair to RNA transcription and processing (3).

Previous studies have shown that PSF is unique among the DBHS proteins for being essential for cell viability in humans and the proper development of T cells and neurons in animal models (7–9). Predictably, mutations and translocations within the PSF gene are common in several diseases ranging from cancers such as leukemia and prostate cancer to neurological disorders like Alzheimer's disease and autism (10–14). Moreover, evidence for direct malfunction of PSF protein has been noted in cases of Alzheimer's and Pick's diseases in which PSF erroneously mislocalizes and accumulates in cytoplasmic inclusions (15). These lines of evidence suggest that PSF activity is critical for normal cell physiology.

PSF's high level of activity in the nucleus is tightly regulated to ensure proper responsiveness to changes in cell state. For example, previous work in our lab has shown that although the abundance of nuclear PSF is unchanged between resting and activated T cells, the ability of PSF to bind to and regulate the CD45 pre-mRNA is dependent on activation of T cell receptor signaling (16). This regulation of PSF's interaction with a target RNA is dependent on the nuclear protein TRAP150 (THRAP3). In unstimulated T cells, GSK3 phosphorylates PSF T687, and this modification promotes TRAP150 binding. The binding of TRAP150 to PSF, in turn, prevents PSF from interacting with the CD45 pre-mRNA. Following T cell receptor activation, GSK3 activity is downregulated and PSF is no longer phosphorylated at T687. As a result, TRAP150 no longer binds PSF, freeing PSF to bind CD45 pre-mRNA and alter its splicing pattern (16). Although TRAP150 clearly influences PSF function, it is not clear how binding of TRAP150 occurs or how binding is related to loss of PSF/RNA interaction. Moreover, only a handful of pre-mRNAs have previously been identified as PSF splicing targets (3). This has prevented a detailed analysis of the scope of PSF's role as a splicing factor and the impact of TRAP150 on this vital nuclear function.

Here, we describe the mechanism underlying TRAP150's effect on PSF's role as a splicing factor. Our data show that TRAP150 forms a minimal intermolecular interface by directly binding PSF's RRMs using a previously uncharacterized 70 residue PSF-interacting domain (PID). Importantly, binding of the PID to PSF's RRMs is sufficient for abrogating interaction with RNA. We also provide evidence that, surprisingly, PSF RRM2, but not RRM1, mediates PSF/RNA contact despite the fact that RRM1 more closely matches the RRM consensus sequence. Finally, we identify ∼40 alternative splicing events in T cells that are sensitive to PSF knockdown, greatly expanding the inventory of genes regulated by PSF. Importantly, many of the validated PSF targets are antagonistically regulated by TRAP150 in the same manner as CD45. Taken together, our results provide greater mechanistic insight into the inhibitory interaction of TRAP150 with PSF, and demonstrate a role for both of these proteins in regulating the alternative splicing of a subset of genes in human T cells.

## MATERIALS AND METHODS

### Cell culture

JSL1 cells were cultured and stimulated as described previously (17). Stable cell lines expressing PSF mutants were produced as described (18). Depletion of PSF was accomplished by lentivirus encoding hairpins targeted to PSF cDNA encoding residues 464–470 (AQKNPMY). Depletion of TRAP150 was performed by antisense morpholino knockdown as previously described (Heyd and Lynch, 2010).

### Protein purification

Cloning for protein expression was accomplished as follows. For stable expression of FLAG-tagged proteins in JSL1 cells, cDNA was inserted downstream of the FLAG tag in the expression vector pEF-nFLAG. For overexpression of TRAP150 in 293 cells, TRAP150 cDNA was cloned into pcDNA3.1 (Life Technologies) downstream of a FLAG tag. For bacterial expression of GST-tagged proteins, cDNA was inserted into the BamHI and EcoRI sites of pGEX-6-P1 (GE Healthcare). For bacterial expression of 6xHis + FLAG-tagged proteins, cDNA was inserted into the NdeI and Xho1 sites of pet15b (Novagen) 3′ to an inserted FLAG tag. For bacterial expression of 6xHis + GB1 tagged proteins, cDNA was cloned into a Gb1 fusion vector pGβ1 courteously provided by Dr. Kevin Gardner.

FLAG-TRAP150 was overexpressed in HEK293 cells using Lipofectamine 2000 (Life Technologies) according to standard procedures. Cells were washed with PBS and lysed by 30 min incubation (on ice) with lysis buffer (25 mM Tris pH 7.5, 150 mM NaCl, 1mM CaCl_2_, 1% v/v Triton X-100 and 1% v/v NP-40). Following centrifugation for 10 min at 17 000 x g, 4°C, lysates were incubated with FLAG-M2 affinity resin (Sigma), washed extensively with TBS and eluted using 3x FLAG peptide. Eluted proteins were dialyzed overnight into storage buffer (20 mM Tris pH 7.5, 300 mM NaCl, 20% v/v glycerol, 1 mM DTT, 0.2 mM EDTA).

For bacterial expression, recombinant proteins were expressed in Rosetta™(DE3)pLysS Competent Cells (Novagen). Overnight starter cultures grown in LB supplemented with ampicillin and chloramphenicol were diluted to A600 = 0.1–0.2 and allowed to grow to A600 = 0.6–0.8 before induction with 1 mM isopropyl β-D-thiogalactopyranoside. Cells were then grown at 37°C for 3–5 h before centrifugation and re-suspension in PMSF-supplemented His binding buffer or PBS following manufacturer's protocols. Cells were lysed by sonication and treated with RNase A, RNase T1 and DNase prior to clarification by centrifugation. His-tagged proteins were isolated by gravity using nickel-nitrilotriacetic acid resin (QIAGEN), and GST-tagged proteins were isolated by gravity using glutathione sepharose 4B resin (GE Healthcare). After extensive washing with His wash buffer or PBS, respectively, tagged-proteins were eluted using buffers supplemented with imidazole or reduced glutathione, respectively. Additional purification was performed by size exclusion chromatography using a Superdex 200 10/300 GL column equilibrated with storage buffer lacking glycerol and eluted at 0.5 ml/min, 25°C. Fractions containing proteins of interest were collected and dialyzed against storage buffer. Amino acids encompassed in the PSF domain deletion mutants are as follows: ΔRRM1, 1–298 + 367–707; ΔRRM2, 1–370 + 450–707; ΔRRMs, 1–298 + 450–707; exRRMs, 266–484; exRRM1, 266–365; exRRM2, 366–484; and minRRMs, 299–449. For ΔRRM1, ΔRRM2 and ΔRRMs, the N- and C- terminal portions of PSF are linked by inclusion of the sequence GGSGHM.

### Circular dichroism

The far-UV spectra of HFexRRM1, HFexRRM2 and HFexRRM1+2 were recorded at 25°C using an Aviv Biomedical model 410 circular dichroism spectrometer. The protein concentration was 25 μM in all experiments, and the buffer conditions were 50 mM phosphate (pH 7.5), 150 mM NaCl for all samples. Spectra shown are the average of 3 scans.

### Co-immunoprecipitation and pulldown assays

Nuclear extracts (NE) were prepared as described (9). For IPs from JSL1 cells, 100 μg of NE, pretreated with RNase A and RNase T1, were incubated with 5 μg PSF antibody (Sigma) or GST antibody (GE Healthcare) in 400 uL IP buffer with rotation (20 mM Tris pH 7.5, 100 mM NaCl, 0.2 mM EDTA, protease inhibitor) overnight at 4°C. Extracts were then incubated for 1 h with protein G Dynabeads (Life Technologies) with rotation. Beads were washed three times with 400 μl IP buffer supplemented with 200 mM NaCl and eluted using 2x Laemmli buffer. Inputs and eluted proteins were then analyzed by western blot. For co-IPs of FLAG-tagged PSF, the above procedure was repeated with NE from JSL1 cells stably expressing FLAG-PSF WT or mutants, with anti-FLAG antibody (Sigma) used to precipitate protein complexes.

For pulldown assays, 40 μg GST-tagged protein was bound to glutathione sepharose 4B beads previously equilibrated and resuspended in 1 ml of PBS for 1.5 h at 4°C with rotation. Bound proteins were washed twice with PBS. Prey proteins were incubated with immobilized bait for 1.5 h at 4°C in 100 μl PBS with rotation. Bound complexes were washed three times with PBS supplemented with 300 mM NaCl before elution in 2x Laemmli buffer. Inputs and eluted proteins were then analyzed by western blot.

### UV Crosslinking and Electrophoretic mobility shift assays (EMSA)

For UV crosslinking assays, 0.03 μg (about 0.5×10^5^ cpm) of uniformly ^32^P-labeled ESS1 RNA was incubated with purified proteins in 10.2 μl reaction volume with final concentrations of 1.3% polyvinyl alcohol, 25 ng/μl of yeast tRNA, 20 ng/μl of BSA, 3 mM MgCl_2_, 1 mM ATP, 20 mM phosphocreatine, 12 mM Tris pH 7.5, 0.1 mM EDTA, 12% glycerol and 120 mM NaCl. For competition, test protein and competitor were pre-incubated for 5 min on ice prior to addition of RNA and further incubation for 20 min, 30°C. Reaction mixtures were crosslinked with 254 nm UV light for 20 min on ice, RNase digested for 20 min at 37°C and analyzed by SDS-PAGE. For EMSAs, proteins were incubated with uniformly labeled RNA adjusted to 1.0 × 10^4^ cpm for 20 min at 30°C in conditions similar to those used in UV crosslinking, excepting the addition of 0.1 μl RNasin (Promega, 40 U/μl), 1 mM DTT and 10 mM KCl. After binding, heparin was added to a final concentration of 0.5 μg/μl and reactions were analyzed on native acrylamide gels (Acrylamide/Bis 29:1, BioRad). RNA Sequences are as follows: *ESS1*-ACGCGUCCACUUUCAAGUGACCCCUUACCUACUCACA CCACUGCAUUCUCACCCGCAAGCACCUUUGACGCGU; *MKK7*- CCUCCUCGUUUAUGAUUUGAUUUCUUUUCUUUUGGACGAAUCGGUCGU UUCUGUUGUGAUUUAUCGUGGUGUUGUUUUUUUCUUCCUUUUCCCCAUCCAG; *SRL-1*- AACCAAGAGGUUUCUCGCGUAUUUCUCUCAUUUUU UUACCCAUUUUACAAAUUUUUUUUGCUAUUUGAGCCAUAGUACCCAUUAAUAGGUCUCGUCCAUUCCCUUGUUUU UUUUUUAUUGUUUCAAUUACACUACAUAAUUAAAAAUCACAUCACUUUCACUCUCACCUUAGUCGUUCUUUAUC AACCAAAAAUAAAAAAAUGCUUCAAUCCGUUGUCUU.

### Antibodies

The following antibodies were used throughout as noted: PSF (Sigma P2860 for IP, Abnova H00006421-A01 for WB), TRAP150 (A300–956A, Bethyl Laboratories), FLAG (2368, Cell Signaling), GST (27–4577–01, GE Healthcare), His (AM1010a, Abgent), hnRNP L (4D11, Abcam), p54^nrb^/NONO (MA3–2024, Affinity Bioreagents), MATR3 (NB100–1761, Novus Biologicals), PSPC1 (a gift from Dr. Archa Fox).

### RASL-Seq and RT-PCR analysis

RASL-Seq was performed as previously described (19,20) using a set of probes that interrogate ∼5,600 specific splicing events. Total RNA was harvested from biologic triplicate samples of wild-type and PSF depleted JSL1 cells grown under normal conditions, or stimulated with PMA. These RNA samples were individually hybridized to the probe set and selected by oligo-dT. Juxtaposed probes annealed to selected RNAs were then ligated and amplified and barcoded by PCR for subsequent multiplexed sequencing on a HiSeq2000. Splicing events were filtered for a minimum of 10 reads averaged across all biologic replicates and conditions and then isoform ratios were calculated by comparing number of reads representing the longest isoform to the number of total reads for that splicing event (PSI = percent spliced in of variable exon). The change in PSI (ΔPSI) was then calculated as the difference between the average PSI across the three biologic replicates of RNA from wildtype cells versus the three replicates of cells depleted of PSF. PMA-induced splicing events that are dependent on PSF were identified as splicing events for which the absolute value of ΔPSI between stimulated WT and stimulated PSF knock-down (KD) cell is ≥10 with a *P*-value < 0.05. Low-cycle RT-PCR to quantify mRNA splicing was done as previously described (17) using ^32^P-labeled primers listed in Supplementary Table S2.

## RESULTS

### The PSF RRMs directly bind TRAP150

**Figure 1.**
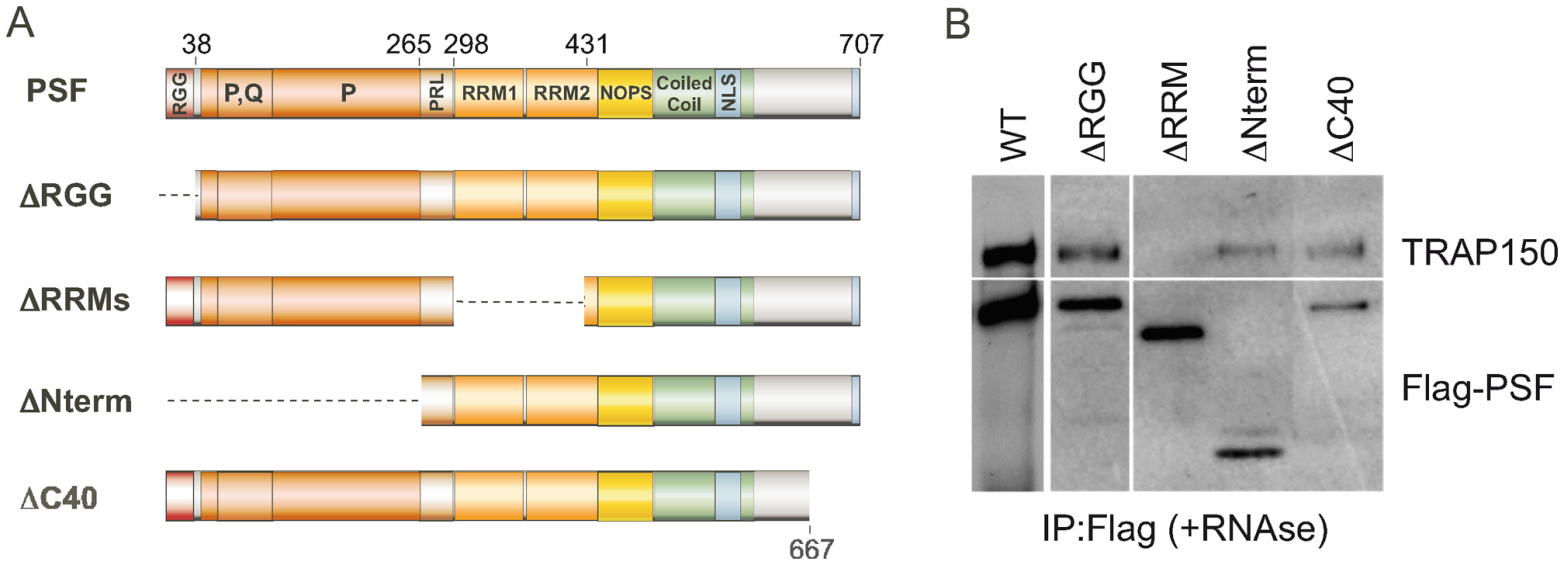
RRMs of PSF are necessary for interaction with TRAP150 in JSL1 cells. (**A**) Schematic of the domain structure of full length PSF and deletion mutants thereof. (**B**) Western blot analysis of co-immunoprecipitations (IP) done from lysates of JSL1 cells expressing the indicated FLAG-tagged versions of PSF. IPs were done using anti-FLAG antibody, and then blotted for FLAG as a loading control, or for endogenous TRAP150.

Previous studies of PSF and TRAP150 have demonstrated that TRAP150 interacts directly with PSF and inhibits the ability of PSF to interact with, and regulate the splicing of, the CD45 pre-mRNA (16). However, the nature of the physical interaction and scope of the functional interplay between TRAP150 and PSF remained unknown. To better characterize the interaction of TRAP150 and PSF, we performed a series of co-immunoprecipitation (co-IP) reactions in nuclear extract from JSL1 Jurkat cells expressing FLAG-tagged PSF deletion mutants (Figure 1). Intriguingly, deletion of most of the tandem RRMs of PSF ablated interaction between PSF and TRAP150 (Figure 1B, ΔRRMs, top), even though removal of the RRMs did not alter nuclear expression (Figure 1B, ΔRRMs, bottom). By contrast, deleting other domains of PSF, including PSF's large N-terminal extension, had no notable impact on PSF's ability to interact with TRAP150 (Figure 1B). Consistent with previous findings (16), the observed interactions were not sensitive to RNase treatment, indicating that the loss of protein–protein interaction observed with the ΔRRMs construct was not due to loss of RNA-binding.

**Figure 2.**
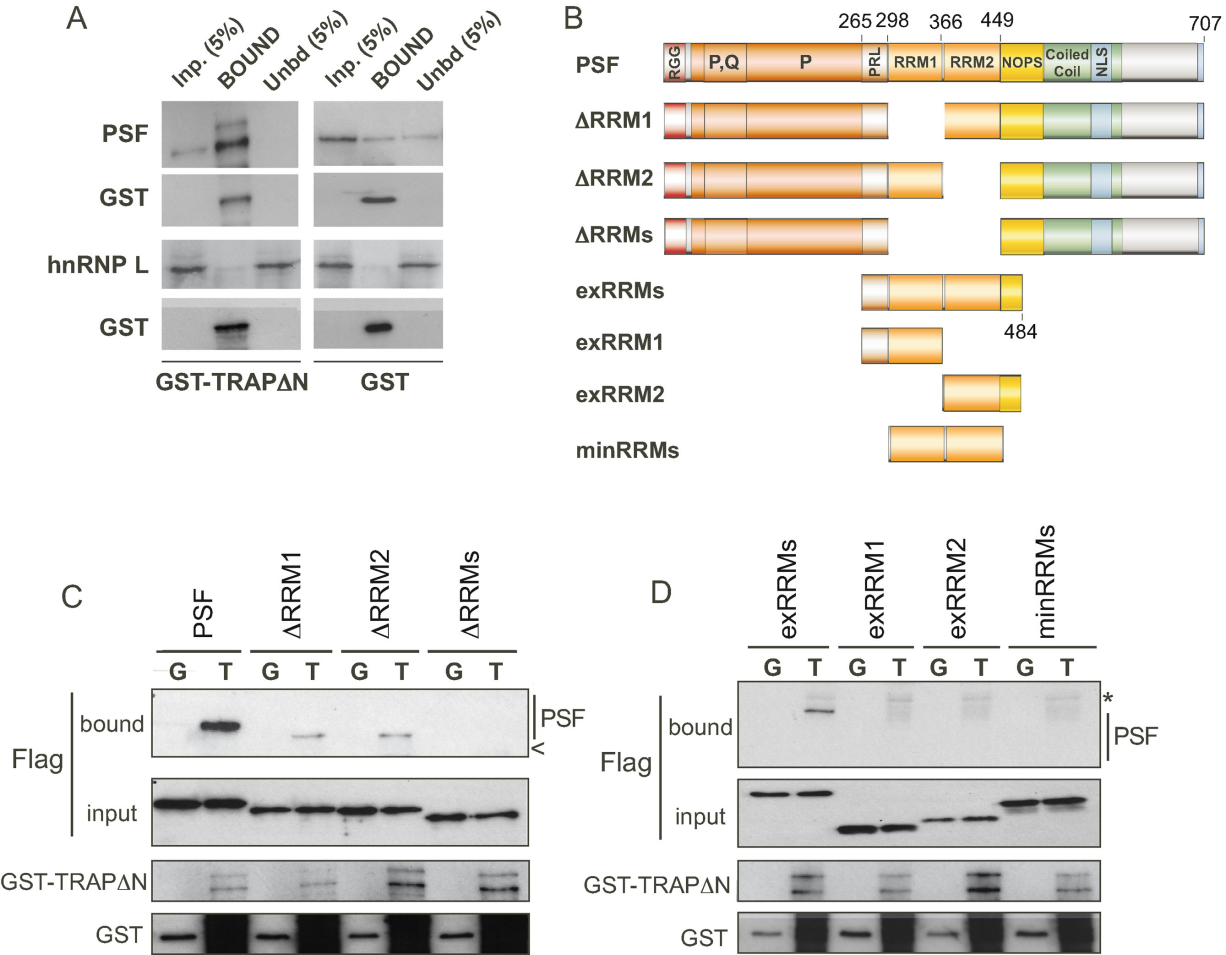
A domain encompassing the RRMs and flanking sequence of PSF is sufficient for interacting directly with TRAP150. (**A**) Western blot analysis of a GST-pull down assay using recombinant GST-tagged TRAP150ΔN (see Figure 3) or GST alone, and full length His-tagged PSF or hnRNP L (as control), all purified from *E. coli*. (**B**) Schematic of additional PSF deletion constructs purified from *E. coli* with N-terminal His and FLAG tags. See Supplemental Figure S1 for analysis of purified proteins. (**C**) Western blot analysis of a GST-pull down assay using recombinant GST-tagged TRAP150ΔN (T) or GST alone (G), and indicated PSF constructs from panel (B). Co-precipitation of PSF (bound) and total (input) was assessed by blotting with anti-FLAG. Arrowhead indicates position where ΔRRMs would migrate. (**D**) Same as panel (C) with more minimal versions of PSF. Asterisk indicates cross-reactivity with a species from the GST-TRAP sample.

In order to directly confirm the role of the PSF RRMs in binding TRAP150, and to eliminate the influence of other potential mammalian cofactors, we next performed a series of GST pulldown assays using recombinant proteins purified from *Escherichia coli*. To facilitate bacterial expression, a truncated form of TRAP150 lacking the N-terminal RS repeats was cloned downstream of a GST-tag to serve as the initial bait (GST-TRAPΔN, Figure 2A). Full-length PSF, and truncations thereof, were purified as N-terminally tandem 6xHis and FLAG (HisFLAG)-tagged fusion proteins (Figure 2B and Supplementary Figure S1). As seen in Figure 2A, GST-TRAPΔN replicated the interaction with full-length PSF observed in mammalian cells, indicating both that TRAP150 and PSF interact directly, and that the first 265 amino acids of TRAP150 are dispensable for this interaction. Importantly, TRAPΔN did not interact with His-hnRNPL, a splicing factor that also regulates CD45 alternative splicing and contains two sets of tandem RRMs (21,22), similar to the RRM arrangement in PSF.

Consistent with the results of the co-IPs from the Jurkat cells, we also found that deletion of both RRMs of PSF ablated the interaction with TRAP150 in this purified system (Figure 2C, ΔRRMs), while deletion of RRM1 or RRM2 alone lessened binding to TRAPΔN (Figure 2C, ΔRRM1 and ΔRRM2). We then tested whether one or both RRMs of PSF were sufficient for interaction with TRAP150. Interestingly, a construct comprising both RRMs along with the N-terminal PR linker and a portion of the NOPS domain showed significant interaction with TRAPΔN (Figure 2D and Supplementary Figure S2A, exRRMs). In contrast, constructs encompassing only RRM1 and the PR-linker region (exRRM1) or RRM2 and a portion of the NOPS domain (exRRM2) both failed to bind GST-TRAPΔN (Figure 2D, Supplementary Figure S2). Additionally, a construct of PSF comprised solely of the dual RRMs with no additional sequence also lacked the ability to bind GST-TRAPΔN (Figure 2D, Supplementary Figure S2B, minRRMs). We note that both RNA–protein interaction studies and circular dichroism indicate that exRRM1, exRRM2 and minRRMs retain secondary structure and are capable of other activities (Supplementary Figures S3 and S4 and see below). Therefore, we conclude that although either RRM is capable of mediating interaction with TRAP150 in the context of the full-length protein, neither RRM alone is a sufficient interface. Instead, interaction with TRAP150 optimally requires both RRMs as well as additional flanking residues of PSF.

### TRAP150 binds PSF using an uncharacterized 70 residue region

**Figure 3.**
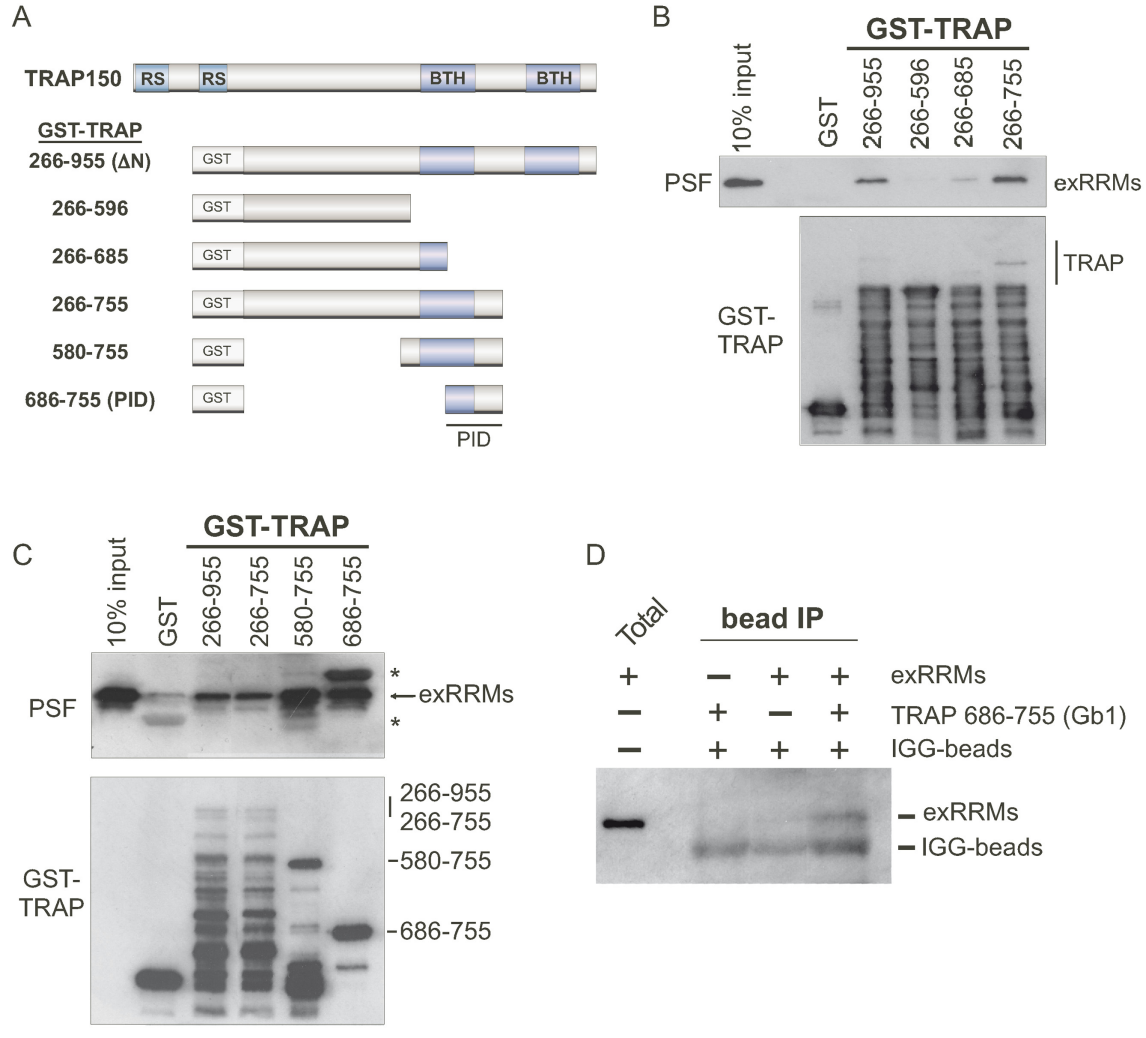
A 70 amino acid region of TRAP150 is sufficient for interacting directly with the exRRMs of PSF. (**A**) Schematic of the known domain structure of full-length TRAP150 and deletion mutants used in study. (**B**) Western blot analysis of GST-pull down assay using the indicated GST-tagged C-terminal deletion versions of TRAP150 (or GST alone) and the His-FLAG purified exRRMs of PSF from Figure 2. Co-precipitation of PSF was assessed using anti-PSF. (**C**) Same as panel (B) but with more minimal versions of TRAP150 as indicated. Asterisks indicate cross-reactivity of the anti-FLAG antibody and GST. (**D**) IP of the exRRMs version of PSF using a Gb1-tagged construct of the TRAP-PID as prey. IgG beads were used to precipitate the Gb1 tag.

Having defined a minimal region of PSF that is sufficient to interact with TRAP150, we next sought to identify the region of TRAP150 responsible for binding PSF. We therefore created a series of GST-tagged TRAP150 mutants for use in GST pulldown assays as above (Figure 3A). First, we made a series of C-terminal truncations based on our initial test construct, TRAPΔN (266–955). Deletion of the most C-terminal 200 amino acids (TRAP266–755) did not reduce interaction with PSF exRRMs; however, a further truncation of 70 amino acids (TRAP266–685) dramatically hindered the ability of TRAP150 to pull-down PSF. No interaction with PSF was observed when an additional 90 amino acids were removed from TRAP (TRAP266–596, Figure 3B). Based on this first round of results, we next generated TRAP150 580–755 and 686–755 to determine if either of these overlapping regions were sufficient for PSF exRRMs interaction (Figure 3C). Indeed, both of these TRAP150 mutants bound the exRRMs. To rule out a tag-related false positive, we repeated the pulldown experiment with a version of TRAP150 686–755 N-terminally tagged with the B1 domain of protein G (Gb1) as bait and HisFLAG-exRRMs as prey, and again observed interaction between these minimal TRAP150 and PSF domains (Figure 3D). In sum, our data indicate that TRAP150 contacts the PSF exRRMs using a PSF-interacting domain (PID) circumscribed by residues 686–755.

### RRM2, but not RRM1, mediates PSF–RNA interaction

**Figure 4.**
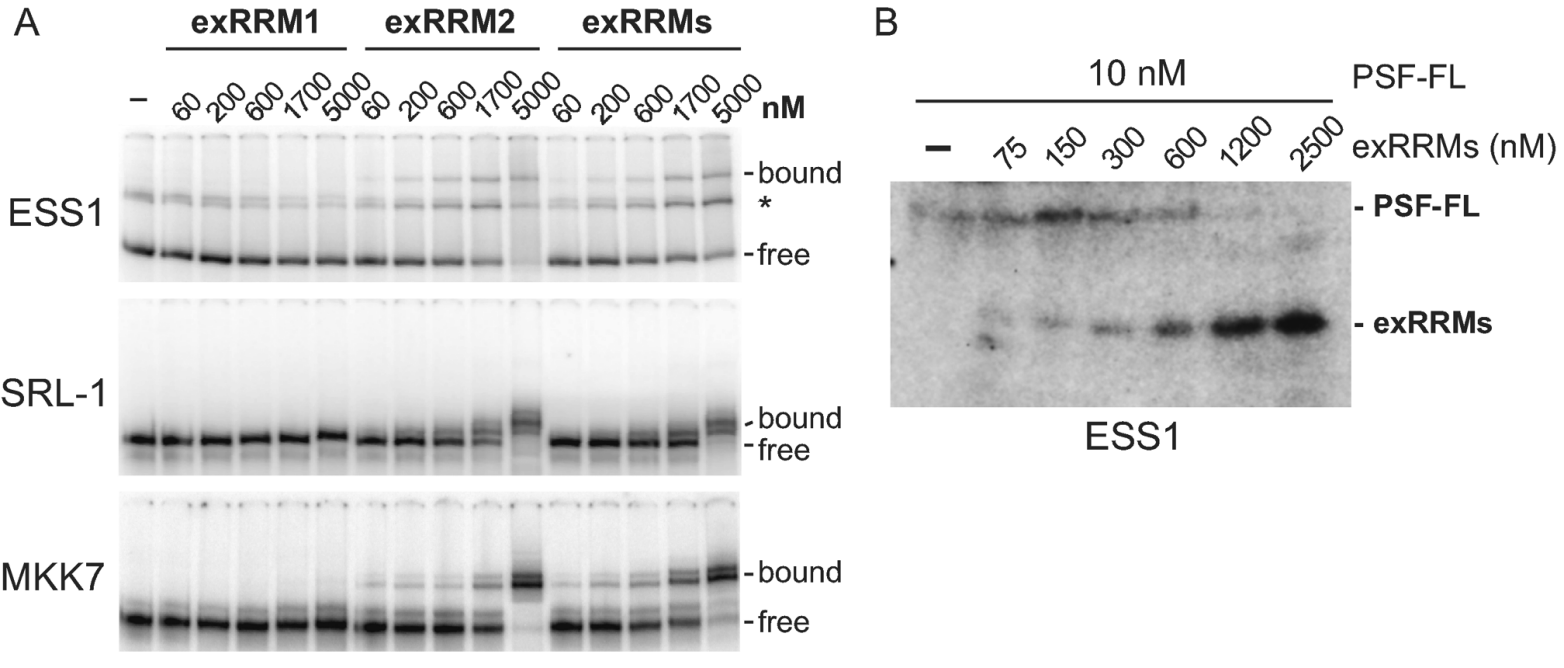
RRM2 of PSF is sufficient for the RRM-mediated RNA-binding activity of PSF. (**A**) Gel shifts of PSF-exRRM1, exRRM2 and exRRMs (see Figure 2B) on three distinct RNAs of differing length and sequence content (see Materials and Methods). Asterisk indicates a species corresponding to structured free RNA. (**B**) UV crosslinking of bacterially expressed and purified full-length PSF and PSF exRRMs on ESS1 RNA.

As mentioned above, we have previously shown that full-length TRAP150 inhibits the binding of full-length PSF to its target sequence on the CD45 pre-mRNA (16). The fact that the PID interacts directly with a region of PSF that encompasses the RRMs suggests that TRAP150 might directly compete with RNA for binding to the same region of PSF. We therefore wanted to determine the minimal region of PSF required for RNA binding. We first compared the relative affinities for RNA of the paired exRRMs to the individual exRRMs using electromobility shift assays (EMSAs). As anticipated, the tandem exRRMs bound readily to the known PSF-target ESS1 RNA from the CD45 RNA (Figure 4A), albeit with lower affinity than the full-length version of PSF based on UV crosslinking competition assays (Figure 4B). The difference in affinity between the exRRMs and full-length PSF is consistent with the fact that sequences beyond the RRMs of PSF have been implicated in nucleic acid binding ((6,23)). More surprising was our observation that exRRM2 bound to the ESS1 RNA with similar affinity as the exRRMs while exRRM1 exhibited little ability to bind ESS1 (Figure 4A, Supplementary Figure S3). This same binding profile for these protein constructs was also observed for additional, unrelated RNAs (Figure 4A, SRL-1, MKK7), with exRRM1 showing unmeasurable affinity for RNA despite containing the canonical RNP motif residues that RRM2 lacks (Figure 4A, Supplementary Figure S3). We note that all three constructs (exRRM1, exRRM2 and exRRMs) produce circular dichromism spectra that are largely similar and likely result from comparable levels of α-helical and β-sheet secondary structure based on the observed ellipticity at 222 nm and 218 nm, respectively (24). It is therefore unlikely that the lack of RNA-binding observed for RRM1 is due to gross loss of secondary structure. (Supplementary Figure S4). Therefore, we conclude that RRM2 mediates the RNA-binding activity of the exRRMs and, similar to full length PSF (3), promiscuously binds a variety of RNA sequences. The fact that RRM2 of PSF lacks a typical RRM sequence configuration suggest that it likely binds RNA by a non-canonical mode, perhaps explaining the promiscuity of RNA binding (see Discussion).

### TRAP150 directly blocks the binding of PSF RRMs to RNA, not protein

**Figure 5.**
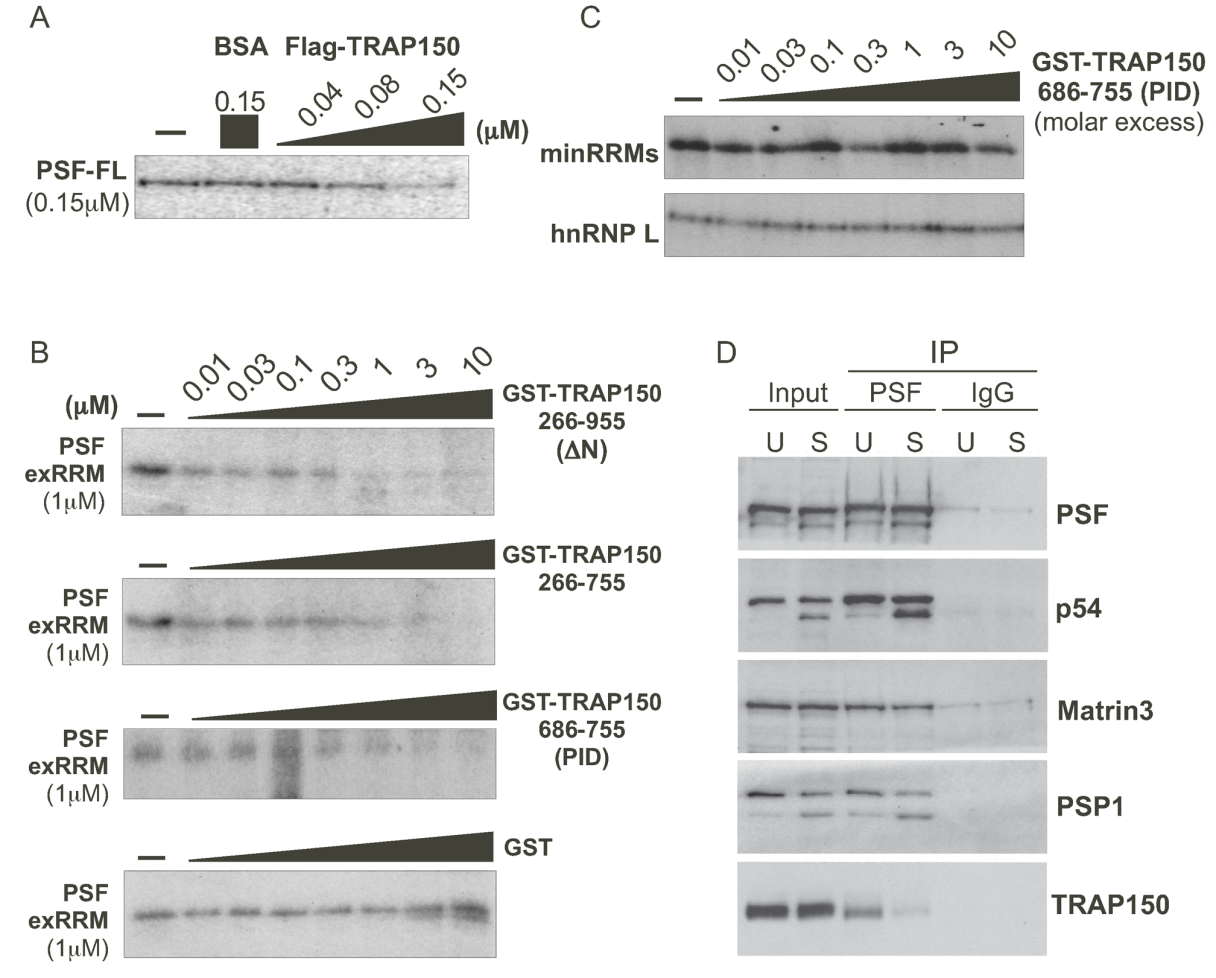
TRAP150 inhibits the RRM-dependent binding activity of PSF, and inhibition of binding requires more than the minimal RRM binding domain. (**A**) UV crosslinking of ESS1 RNA with full-length PSF (PSF-FL) either alone (-), in the presence of BSA as a control or in the presence of increasing amounts of full-length FLAG-tagged TRAP150. (**B**) Same as panel (A), but competing binding of exRRMs of PSF with indicated truncations of GST-TRAP150 or GST alone. (**C**) Same as panel (A) but competing for binding of the minRRMs and hnRNP L by GST-TRAP(PID). (**D**) Western blots of immunoprecipitation of PSF from unstimulated (TRAP150 bound) and stimulated (TRAP150 unbound) JSL1 cells showing relative binding of TRAP150 and other known PSF-interacting partners. The source of the doublet for PSPC1 and p54^nrb^/NONO in stimulated cells is unknown, but is the same in input and IP samples.

Having shown that the exRRMs are sufficient for both RNA binding and interaction with TRAP150, we wanted to determine if the minimal interaction of TRAP150 and PSF is sufficient for the inhibition of the PSF–RNA interaction observed with the full-length proteins ((16); Figure 5A). Using UV crosslinking competition assays, we found that both the N- and C- termini of TRAP150 were dispensable for blocking the interaction of the exRRMs of PSF with RNA (Figure 5B, TRAP150-ΔN and 266–755). Indeed, even the minimal TRAP150 PID polypeptide blocked exRRM/RNA interaction efficiently, while the GST tag alone had no significant impact on the interaction of the exRRMs with RNA. We note that in all cases we saw nearly complete inhibition of PSF/RNA interactions when TRAP150 was equimolar to PSF, suggesting that inhibition of PSF/RNA binding is achieved through a complex between TRAP150 and PSF with one-to-one stoichiometry. Moreover, efficient interaction by TRAP150 is required for its ability to inhibit RNA binding, as the TRAP150 PID did not inhibit RNA binding of either the minRRMs of PSF or hnRNP L (Figure 5C), neither of which interact with the PID (Supplementary Figure S5).

PSF is known to use its RRM domains not only to bind RNA, but also to bind other proteins, especially other DBHS proteins (3). Therefore, we also tested if TRAP150 disrupts PSF protein/protein interactions. To best replicate physiologically relevant protein–protein interactions, we performed these assays in cell lysates, using cell stimulation to control TRAP150-PSF interaction. As previously reported, TRAP150 binds efficiently to PSF in unstimulated Jurkat cells, but much less so when these cells are stimulated with PMA (phorbol myristate acetate) ((16), Figure 5D), providing a convenient way to compare PSF complex formation in the presence or absence of TRAP150. Intriguingly, we observed no difference in the efficiency with which the DBHS proteins p54^nrb^/NONO and PSPC1, or the nuclear matrix protein Matrin 3, co-precipitated with PSF from stimulated (S) versus unstimulated (U) cells, despite a marked difference in TRAP150 co-precipitation (Figure 5C). Therefore, we conclude that the interaction of TRAP150 with PSF neither promotes nor hinders the ability of PSF to interact with other proteins via its RRM domains. These data indicate that the functional regulation of PSF by TRAP150 is primarily through modulation of PSF's RNA-binding activity.

### PSF and TRAP150 broadly regulate alternative splicing in JSL1 cells

**Figure 6.**
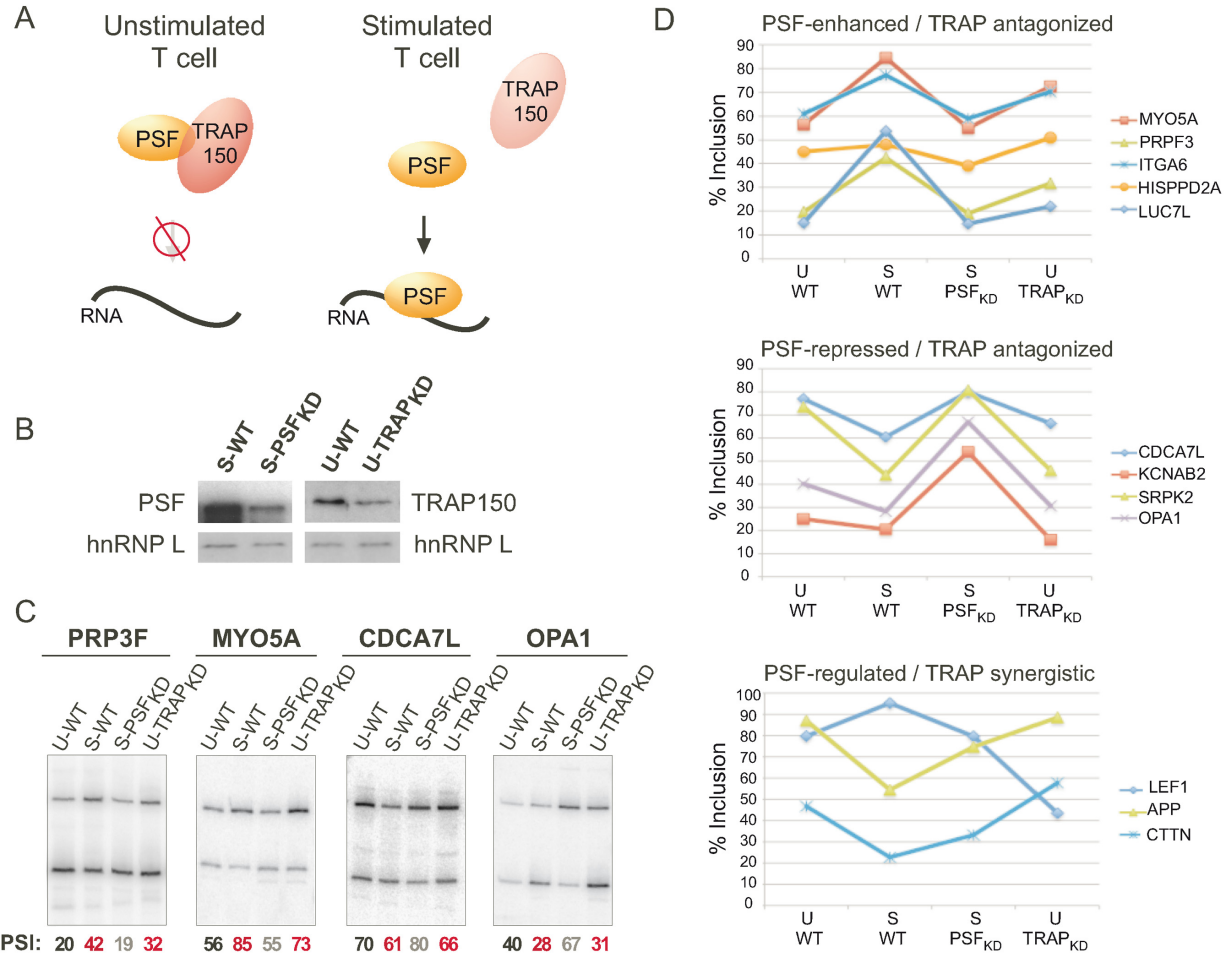
Expression of TRAP150 antagonizes the majority of PSF-dependent splicing events. (**A**) Model of TRAP150 regulation of PSF in unstimulated and stimulated T cells based on studies of the CD45 pre-mRNA. (**B**) Western blots showing knock-down of TRAP150 in resting cells (to simulate stimulated conditions) and knock-down of PSF in stimulated cells (to simulate unstimulated conditions). (**C**) Representative RT-PCR to assay inclusion of PSF-target exons in cell conditions shown in panel (B). (**D**) Graphical representation of inclusion of variable exons in the indicated genes in unstimulated or stimulated wildtype and PSF or TRAP150-depleted cells. Top graph are exons that are enhanced by PSF in stimulated cells and repressed by TRAP150 in unstimulated cells. The middle graph are exons that are repressed by PSF in stimulated cells and enhanced by TRAP150 in unstimulated cells. The bottom graph are exons that are regulated in the same direction by both PSF and TRAP150. In all cases %inclusion is derived from low-cycle RT-PCR and is the average of at least three independent experiments. Standard deviation in all cases is ≤ ±5.

Despite the fact that T cell stimulation controls the interaction of TRAP150 with PSF, and the interaction with TRAP150 regulates RNA binding by PSF (Figure 6A), only one alternative splicing event has thus far been shown to be controlled by this regulatory circuit (3,16). To determine if PSF/TRAP150 have a broader impact on splicing in T cells, we used a variant of RNA-Seq that queries a set of ∼5600 alternative splicing events (RASL-Seq, (19,20)). We first identified exons that are regulated upon PMA stimulation in a manner that is dependent on PSF by comparing RNA isolated from unstimulated (U) and stimulated (S) wildtype (WT) cells to RNA isolated from cells depleted of PSF by shRNA knockdown (Supplementary Table S1, Figure 6B). Specifically, we identified exons that exhibited a change in inclusion in wildtype cells (difference in Percent Spliced Isoform (PSI) of at least 9, |ΔPSI|>9) between unstimulated and stimulated conditions, and for which depletion of PSF in stimulated cells resulted in a significant PSI value similar to that observed in the wildtype unstimulated cells (PSI_WT-S_ − PSI_PSF-S_ ≥ (0.6) PSI_WT-S_ − PSI_WT-U_, *P* < 0.05) (Table 1, Supplementary Table S1). In total, 39 alternative splicing events were sensitive to PSF knockdown by this criteria (Table 1). From this set, 16 were assayed by RT-PCR, with 12 showing marked changes upon PSF depletion and another 4 showing modest effects consistent with PSF-regulation (Figure 6C, D).

**Table 1.** PSF-dependent PMA induced splicing events (determined by RASL-Seq)

	Percent Spliced Isoform		
Gene symbol	WT Unstim	WT Stim	PSF KD Stim
LEF1	36	88	48
DKFZp434I0612	46	61	24
LUC7L	31	66	32
AMOTL1	70	86	58
BAT2L	8	38	11
MYO5A	59	77	54
PRPF3	18	41	19
TRA2A	33	50	32
PPIL5	34	56	40
NFYA	5	28	15
BBX	14	25	10
SNHG3-RCC1	36	55	42
ITGA6	34	44	32
C20orf72	80	70	82
HISPPD2A	26	9	21
USP33	33	21	35
KLC1	93	77	91
HNRNPH3	78	64	80
ATP11C	44	32	48
GNAS	73	62	80
MATR3	80	68	85
SESTD1	67	33	51
C10orf28	42	32	53
PEX5L	87	77	96
WHSC1L1	40	29	49
NCOR2	81	66	86
CCDC7	53	38	61
ADNP	84	69	92
DTNB	27	12	36
CDCA7L	37	21	48
CTTN	73	43	70
SNHG3-RCC1	44	25	53
APP	89	63	92
C2orf33	46	25	56
RPGR	76	64	95
TPIP	80	59	92
KCNAB2	33	22	57
SRPK2	48	32	68
OPA1	52	33	75

Finally, to evaluate the impact of TRAP150 on PSF splicing targets, we next examined robust PSF targets for sensitivity to TRAP150 knockdown. We specifically focused on TRAP150's impact in resting cells, as knockdown of TRAP150 should force a stimulated-like state for PSF targets based on the PSF/TRAP150 dynamic observed for CD45 (Figure 6A). Fitting this model of regulation, most PSF-responsive genes exhibited both (i) sensitivity to TRAP150 levels, and (ii) changes in exon inclusion consistent with TRAP150 knockdown promoting stimulated-like PSF activity (Figure 6B–D). For example, stimulation of JSL1 cells promoted PRP3F exon inclusion at wild type PSF levels, but this effect was lost when PSF was knocked down (Figure 6C). Correspondingly, knockdown of TRAP150 in resting cells raised exon inclusion to levels approaching what was seen in stimulated cells (Figure 6C, D). This antagonistic regulation was also seen for several PSF-repressed exons (Figure 6C, D, middle), similar to what has been shown for CD45 (16). Only 3 of the 12 exons tested showed an effect of TRAP150 depletion different from that predicted by the model of TRAP150/PSF antagonism (Figure 6D, bottom). Therefore, we conclude that PSF regulates the splicing of multiple exons in T cells in a stimulation-dependent manner, and that TRAP150 regulates the majority of these PSF-dependent events in a manner consistent with it inhibiting PSF activity in resting T cells.

## DISCUSSION

Here, we identify a minimal PSF-interacting domain (PID) of TRAP150, and demonstrate that this TRAP150 PID binds directly to PSF's RNA-binding domain and inhibits the interaction of PSF with pre-mRNA. We also show that RRM2, but not RRM1, underpins PSF binding to RNA. Finally, we use RASL-Seq to uncover dozens of novel PSF alternative splicing targets in T cells and find that TRAP150 antagonizes PSF's influence on the majority of these exon splicing targets in a manner consistent with CD45-type regulation. This study therefore deepens our understanding of TRAP150's role as a negative regulator of PSF function and broadens our view of the role of these proteins in alternative splicing in T cells.

Our previous findings showed that TRAP150 tightly controls PSF's contributions to CD45 alternative splicing regulation in T cells (16); however, it was unclear how TRAP150 exerts this control. Work from others has suggested that PSF in cells exists as part of obligate homo- or heterodimers with the other DBHS proteins (25,26). These dimers are held together in large part because of the tight interface formed by RRM2 of one monomer and the NOPS domain of the counterpart monomer (∼65% of the total dimer interface) (5,6). Our results here show that the TRAP150 PID binds directly to a sequence encompassing the RRMs and NOPS domains of PSF (Figures 2 and 3, Supplementary Figure S2) and that TRAP150 interaction with PSF is not exclusive of interactions between PSF and the other DBHS proteins, p54^nrb^/NONO and PSPC1 (Figure 5D). These data suggest a plausible model for TRAP150 interaction with PSF in which TRAP150 recognizes PSF homo- or heterodimers as the basic unit of interaction and specifically prevents RNA interaction with RRM2 by docking onto the dimers. In this model, the lack of PID interaction with the minRRMs would be predicted as a consequence of decreased dimerization caused by NOPS domain truncation (5,6). Moreover, interaction of TRAP150 across the dimer interface would likely occlude access to the 20 Å, solvent-filled channel that is bounded by the RRM2 of each dimer partner (5,6). Based on our finding that RRM2 binds mRNA, this channel is potentially the site of RNA binding, thereby explaining how TRAP150 interaction precludes RNA association. Since we observe RNA binding inhibition at equal molar concentration of TRAP150 and PSF, we predict that RNA binding is maximally inhibited when a monomer of TRAP150 binds across each face of the symmetric dimer in a 2:2 stoichiometry. We do note, however, we cannot rule out alternative model(s), including the possibility that binding of TRAP150 to PSF promotes some allosteric change that inhibits RNA association. We also note that our work thus far does not provide an explanation for why phosphorylation of PSF T687 promotes interaction with TRAP150. One potential model, however, is that the low complexity C-terminus of PSF, when dephosphorylated, forms an intramolecular interaction with the exRRMs region that is mutually exclusive with the TRAP150 interaction.

Importantly, regardless of the actual mechanism of TRAP150 inhibition of PSF, our results add to a growing body of evidence that suggests that PSF function is regulated through cell state-specific competition among protein, RNA and DNA interactors (3). For instance, NEAT1 and MALAT1 ncRNAs regulate PSF's ability to bind transcription targets or PTB2, respectively, in regulatory regimes highly dependent on cellular expression of those RNAs (27–29). Thus the relative expression of cofactors versus targets is likely critical for fine-tuning the nuclear activity of PSF. In this way, TRAP150 may specifically alter some or all RNA binding-dependent functions while allowing other PSF functions, such as DNA damage repair, though more study will be required to explore these possibilities.

One crucial facet of PSF function that has been underexplored is the scope of PSF's role as a splicing factor. Although PSF was first identified in the context of constitutive splicing, and has since been examined as an alternative splicing regulator, only a handful of genes have been identified or examined as targets of PSF splicing factor function (3). Our identification by RASL-Seq of nearly 40 splicing events subject to PSF-dependent regulation in T cells dramatically expands the catalog of genes regulated by PSF. Furthermore, we show that the vast majority of genes sensitive to PSF knockdown also are sensitive to TRAP150 knockdown. Specifically, we find that loss of TRAP150 causes a splicing response similar to a change in cell state from unstimulated to stimulated cells, consistent with both T cell stimulation and TRAP150 depletion freeing PSF from TRAP150-mediated sequestration (Figure 6). These results indicate that the PSF-TRAP150 interaction we characterize here has a broader impact on splicing than simply the CD45 gene. Indeed, as our detection of PSF/TRAP150-sensitive splicing is limited to those within the ∼5600 splicing events interrogated by RASL-Seq, we predict that the genes we identify here are an underestimate of the splicing events controlled by these proteins.

Finally, this report adds to our knowledge of TRAP150, an understudied protein that nevertheless seems be quite active in the nucleus. Previous studies of TRAP150 have demonstrated a role in the DNA damage response, mRNA degradation and subnuclear distribution in addition to its function as a splicing factor in its own right (30–35). However, little is known about how TRAP150 biochemically mediates this set of functions. Lee and colleagues have shown that TRAP150's RS repeats are necessary for its role in splicing activation, while residues 596–955 contribute in some fashion to noncanonical mRNA degradation (34). We contribute to the model of TRAP150 as a modular protein by showing that the TRAP150 PID (residues 686–755) interacts with PSF and concomitantly blocks PSF/RNA interaction. It will be interesting to explore whether this PID domain interacts with other nuclear factors as a general protein–protein interacting domain or if it contributes to other roles for TRAP150 in the nucleus. In addition, further exploration of TRAP150's role in regulating PSF's splicing factor function, and the mechanism underlying the phosphorylation-dependent shift in PSF's affinity for TRAP150 (16), will be important to provide continued insight into the broad regulation of gene expression in T cells.

## Supplementary Material

SUPPLEMENTARY DATA

## References

[1] Fu X.D., Ares M. Jr (2014). Context-dependent control of alternative splicing by RNA-binding proteins. Nat. Rev. Genet..

[2] Gerstberger S., Hafner M., Ascano M., Tuschl T. (2014). Evolutionary conservation and expression of human RNA-binding proteins and their role in human genetic disease. Adv. Exp. Med. Biol..

[3] Yarosh C.A., Iacona J.R., Lutz C.S., Lynch K.W. (2015). PSF: nuclear busy-body or nuclear facilitator. Wiley Interdiscip. Rev. RNA.

[4] Fox A.H., Lamond A.I. (2010). Paraspeckles. Cold Spring Harb. Perspect. Biol..

[5] Passon D.M., Lee M., Rackham O., Stanley W.A., Sadowska A., Filipovska A., Fox A.H., Bond C.S. (2012). Structure of the heterodimer of human NONO and paraspeckle protein component 1 and analysis of its role in subnuclear body formation. Proc. Natl. Acad. Sci. U.S.A..

[6] Lee M., Sadowska A., Bekere I., Ho D., Gully B.S., Lu Y., Iyer K.S., Trewhella J., Fox A.H., Bond C.S. (2015). The structure of human SFPQ reveals a coiled-coil mediated polymer essential for functional aggregation in gene regulation. Nucleic Acids Res..

[7] Lowery L.A., Rubin J., Sive H. (2007). Whitesnake/sfpq is required for cell survival and neuronal development in the zebrafish. Dev. Dyn..

[8] Heyd F., Lynch K.W. (2011). PSF controls expression of histone variants and cellular viability in thymocytes. Biochem. Biophys. Res. Commun..

[9] Melton A.A., Jackson J., Wang J., Lynch K.W. (2007). Combinatorial control of signal-induced exon repression by hnRNP L and PSF. Mol. Cell. Biol..

[10] Jiang F.N., He H.C., Zhang Y.Q., Yang D.L., Huang J.H., Zhu Y.X., Mo R.J., Chen G., Yang S.B., Chen Y.R. (2013). An integrative proteomics and interaction network-based classifier for prostate cancer diagnosis. PLoS One.

[11] Duhoux F.P., Auger N., De Wilde S., Wittnebel S., Ameye G., Bahloula K., Van den Berg C., Libouton J.M., Saussoy P., Grand F.H. (2012). The t(1;9)(p34;q34) fusing ABL1 with SFPQ, a pre-mRNA processing gene, is recurrent in acute lymphoblastic leukemias. Leuk. Res..

[12] Dolnik A., Engelmann J.C., Scharfenberger-Schmeer M., Mauch J., Kelkenberg-Schade S., Haldemann B., Fries T., Kronke J., Kuhn M.W., Paschka P. (2012). Commonly altered genomic regions in acute myeloid leukemia are enriched for somatic mutations involved in chromatin remodeling and splicing. Blood.

[13] Mathur M., Samuels H.H. (2007). Role of PSF-TFE3 oncoprotein in the development of papillary renal cell carcinomas. Oncogene.

[14] Stamova B.S., Tian Y., Nordahl C.W., Shen M.D., Rogers S., Amaral D.G., Sharp F.R. (2013). Evidence for differential alternative splicing in blood of young boys with autism spectrum disorders. Mol. Autism..

[15] Ke Y.D., Dramiga J., Schutz U., Kril J.J., Ittner L.M., Schroder H., Gotz J. (2012). Tau-mediated nuclear depletion and cytoplasmic accumulation of SFPQ in Alzheimer's and Pick's disease. PLoS One.

[16] Heyd F., Lynch K.W. (2010). Phosphorylation-dependent regulation of PSF by GSK3 controls CD45 alternative splicing. Mol. Cell.

[17] Lynch K.W., Weiss A. (2000). A model system for the activation-induced alternative-splicing of CD45 implicates protein kinase C and Ras. Mol. Cell. Biol..

[18] Rothrock C., Cannon B., Hahm B., Lynch K.W. (2003). A conserved signal-responsive sequence mediates activation-induced alternative splicing of CD45. Mol. Cell.

[19] Li H., Qiu J., Fu X.D. (2012). RASL-seq for massively parallel and quantitative analysis of gene expression. Curr. Protoc. Mol. Biol..

[20] Mallory M.J., Allon S.J., Qiu J., Gazzara M.R., Tapescu I., Martinez N.M., Fu X.D., Lynch K.W. (2015). Induced transcription and stability of CELF2 mRNA drives widespread alternative splicing during T-cell signaling. Proc. Natl. Acad. Sci. U.S.A..

[21] Blatter M., Dunin-Horkawicz S., Grishina I., Maris C., Thore S., Maier T., Bindereif A., Bujnicki J.M., Allain F.H. (2015). The Signature of the Five-Stranded vRRM Fold Defined by Functional, Structural and Computational Analysis of the hnRNP L Protein. J. Mol. Biol..

[22] Rothrock C.R., House A.E., Lynch K.W. (2005). HnRNP L represses exon splicing via a regulated exonic splicing silencer. EMBO J..

[23] Kramer K., Sachsenberg T., Beckmann B.M., Qamar S., Boon K.L., Hentze M.W., Kohlbacher O., Urlaub H. Photo-cross-linking and high-resolution mass spectrometry for assignment of RNA-binding sites in RNA-binding proteins. Nat. Methods.

[24] Kelly S.M., Jess T.J., Price N.C. (2005). How to study proteins by circular dichroism. Biochim. Biophys. Acta.

[25] Kuwahara S., Ikei A., Taguchi Y., Tabuchi Y., Fujimoto N., Obinata M., Uesugi S., Kurihara Y. (2006). PSPC1, NONO, and SFPQ are expressed in mouse Sertoli cells and may function as coregulators of androgen receptor-mediated transcription. Biol. Reprod..

[26] Fox A.H., Bond C.S., Lamond A.I. (2005). P54nrb forms a heterodimer with PSP1 that localizes to paraspeckles in an RNA-dependent manner. Mol. Biol. Cell.

[27] Ji Q., Zhang L., Liu X., Zhou L., Wang W., Han Z., Sui H., Tang Y., Wang Y., Liu N. (2014). Long non-coding RNA MALAT1 promotes tumour growth and metastasis in colorectal cancer through binding to SFPQ and releasing oncogene PTBP2 from SFPQ/PTBP2 complex. Br. J. Cancer.

[28] Hirose T., Virnicchi G., Tanigawa A., Naganuma T., Li R., Kimura H., Yokoi T., Nakagawa S., Benard M., Fox A.H. (2014). NEAT1 long noncoding RNA regulates transcription via protein sequestration within subnuclear bodies. Mol. Biol. Cell.

[29] Imamura K., Imamachi N., Akizuki G., Kumakura M., Kawaguchi A., Nagata K., Kato A., Kawaguchi Y., Sato H., Yoneda M. (2014). Long noncoding RNA NEAT1-dependent SFPQ relocation from promoter region to paraspeckle mediates IL8 expression upon immune stimuli. Mol. Cell.

[30] Bracken C.P., Wall S.J., Barre B., Panov K.I., Ajuh P.M., Perkins N.D. (2008). Regulation of cyclin D1 RNA stability by SNIP1. Cancer Res..

[31] Merz C., Urlaub H., Will C.L., Luhrmann R. (2007). Protein composition of human mRNPs spliced in vitro and differential requirements for mRNP protein recruitment. RNA.

[32] Lee K.M., Tarn W.Y. (2014). TRAP150 activates splicing in composite terminal exons. Nucleic Acids Res..

[33] Varia S., Potabathula D., Deng Z., Bubulya A., Bubulya P.A. (2013). Btf and TRAP150 have distinct roles in regulating subcellular mRNA distribution. Nucleus.

[34] Lee K.M., Hsu I.A. W., Tarn W.Y. (2010). TRAP150 activates pre-mRNA splicing and promotes nuclear mRNA degradation. Nucleic Acids Res..

[35] Beli P., Lukashchuk N., Wagner S.A., Weinert B.T., Olsen J.V., Baskcomb L., Mann M., Jackson S.P., Choudhary C. (2012). Proteomic investigations reveal a role for RNA processing factor THRAP3 in the DNA damage response. Mol. Cell.

