# Supplementary material for: TRAP150 interacts with the RNA-binding domain of PSF and antagonizes splicing of numerous PSF-target genes in T cells
Source: Nucleic Acids Res. 2015 Oct 10;43(18):9006–16. doi: 10.1093/nar/gkv816 (PMC4605305; doi:10.1093/nar/gkv816)
Supplement: SUPPLEMENTARY DATA [file supp_43_18_9006__index.html]

TRAP150 interacts with the RNA-binding domain of PSF and antagonizes splicing of numerous PSF-target genes in T cells — TRAP150 interacts with the RNA-binding domain of PSF and antagonizes splicing of numerous PSF-target genes in T cells — SUPPLEMENTARY DATA 

# TRAP150 interacts with the RNA-binding domain of PSF and antagonizes splicing of numerous PSF-target genes in T cells

## SUPPLEMENTARY DATA

- SUPPLEMENTARY DATA
- SUPPLEMENTARY DATA
