# Supplementary material for: TRAP150 interacts with the RNA-binding domain of PSF and antagonizes splicing of numerous PSF-target genes in T cells
Source: Nucleic Acids Res. 2015 Oct 10;43(18):9006–16. doi: 10.1093/nar/gkv816 (PMC4605305; doi:10.1093/nar/gkv816)
Supplement: SUPPLEMENTARY DATA [file supp_gkv816_nar-01883-a-2015-File008.pdf]

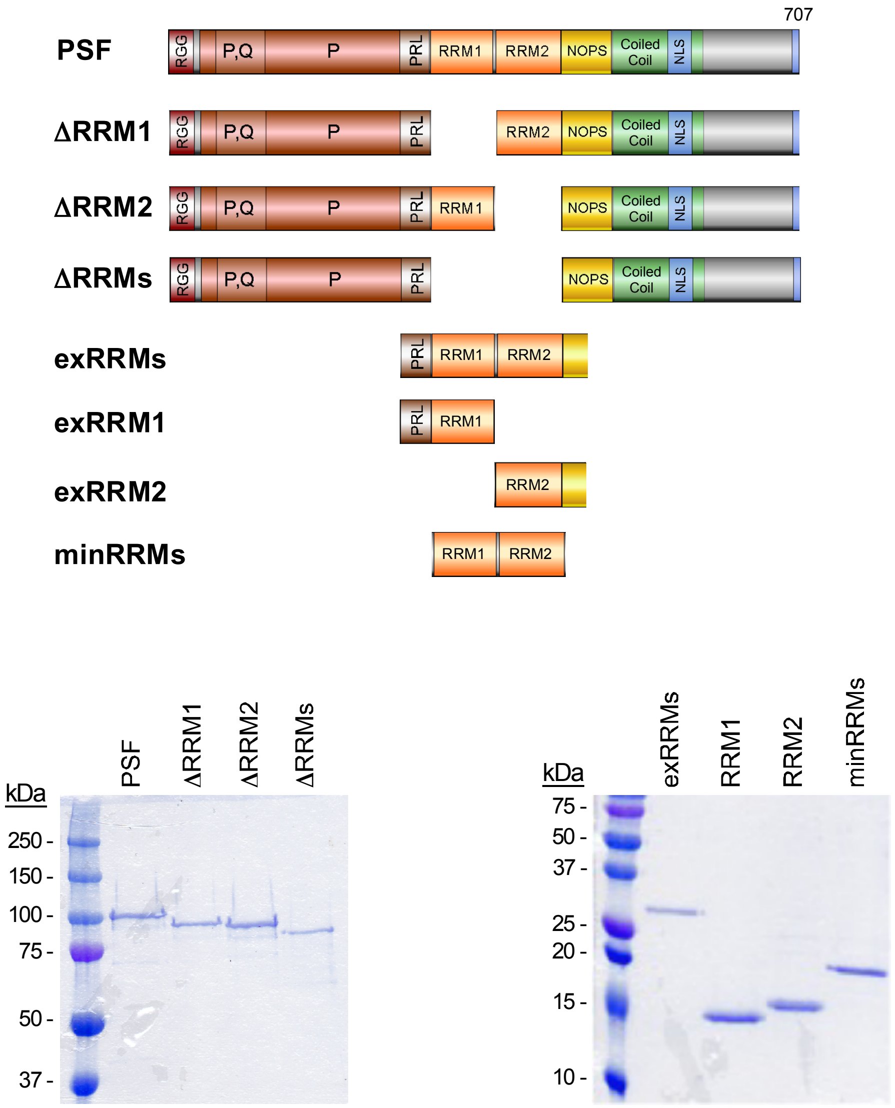

Figure S1: Purified PSF constructs used for pulldown assays in Figures 2 and 3. Schematics of the domain structure of full length PSF and deletion mutants of proteins purified for use in this study. Coomassie stains are of 1 μg of each construct as determined by Bradford assay following purification and dialysis as described in the Materials and Methods.

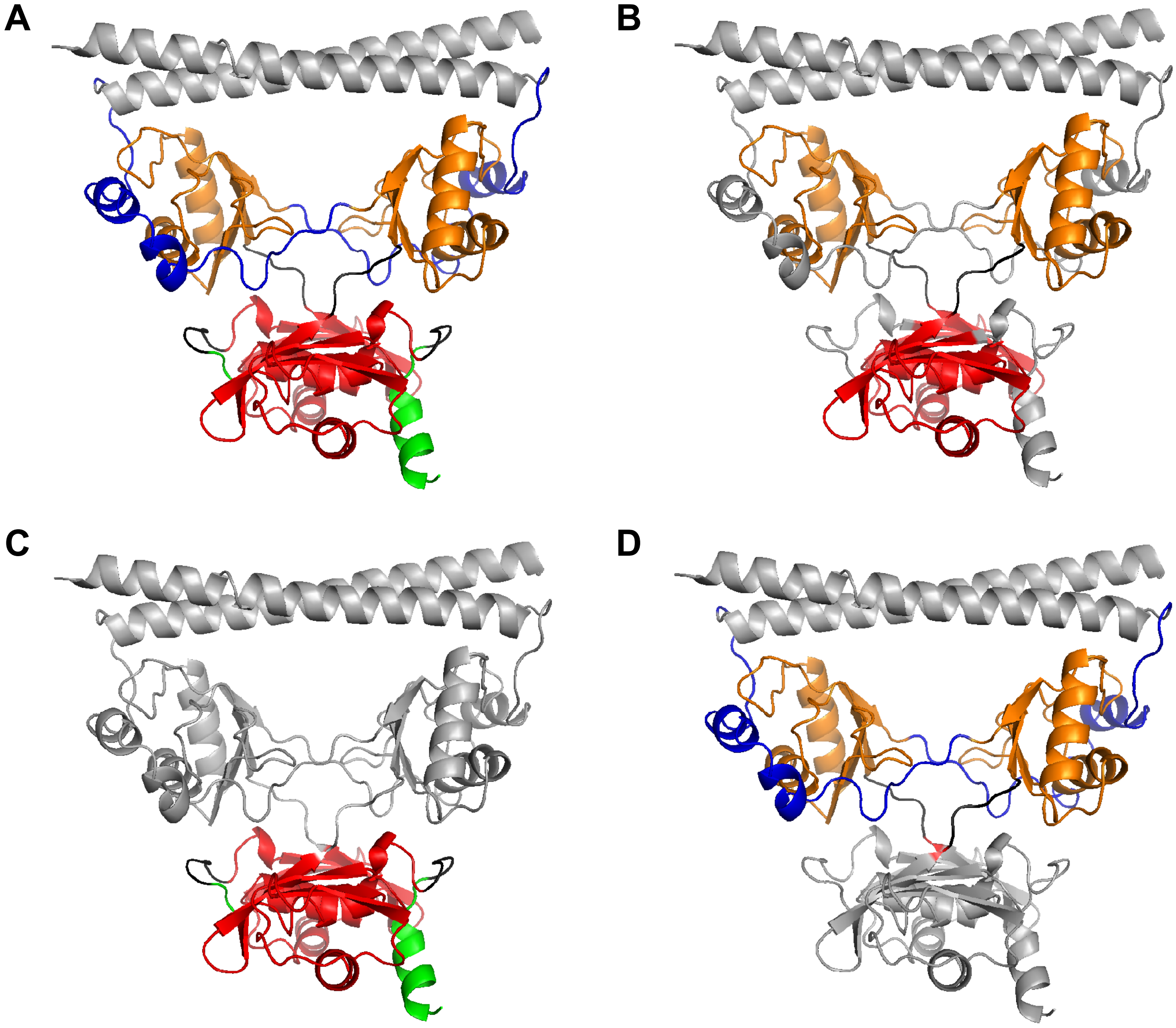

Figure S2: PSF domains modeled on published structure. The published structure of a PSF homodimer (ref 6, PDB 4WII.A) is shown with colored portion indicating that which is encompassed in the (A) exRRMs, (B) minRRMs, (C) exRRM1 and (D) exRRM2 constructs used in this study. Colors are as follows: red, RRM1; orange, RRM2; blue, NOPS; green, N-terminal extension from RRM1 (a portion of what is referred to as “DNA binding domain (ref 6) or PRL (ref 3)). Grey indicates sequences outside each of the contstructs. Note that an additional 10 N-terminal amino acids are present in the exRRMs and exRRM1 constructs that are not included in the published structure.

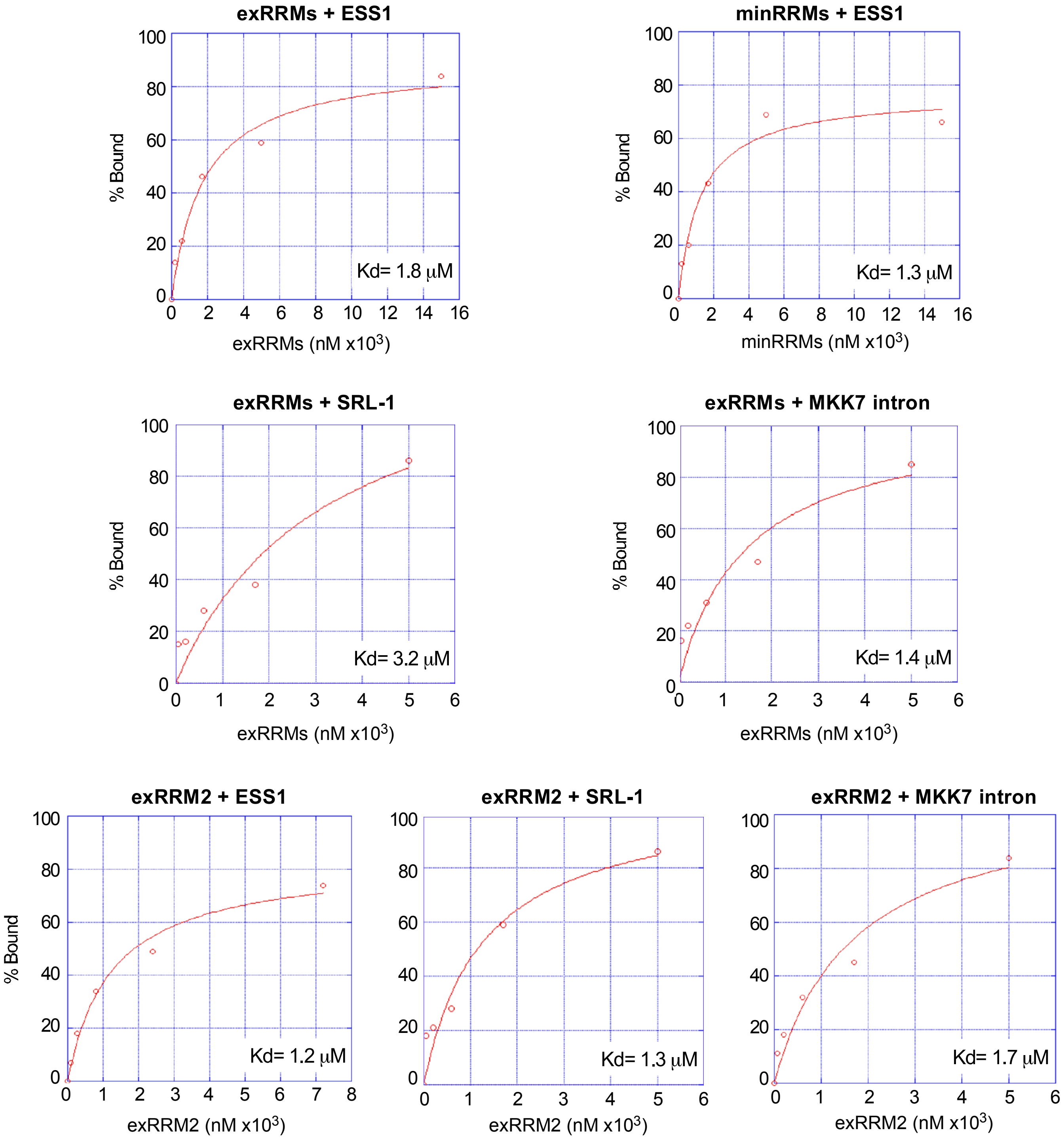

Figure S3: Graphs used to determine relative dissociation constants (K_d) for PSF RRM constructs. EMSAs were performed in triplicate as described in Materials and Methods for each of the constructs and probes listed. Data were plotted and fit with a nonlinear least squares regression using Kaleidagraph (Synergy) to obtain values for relative K_d

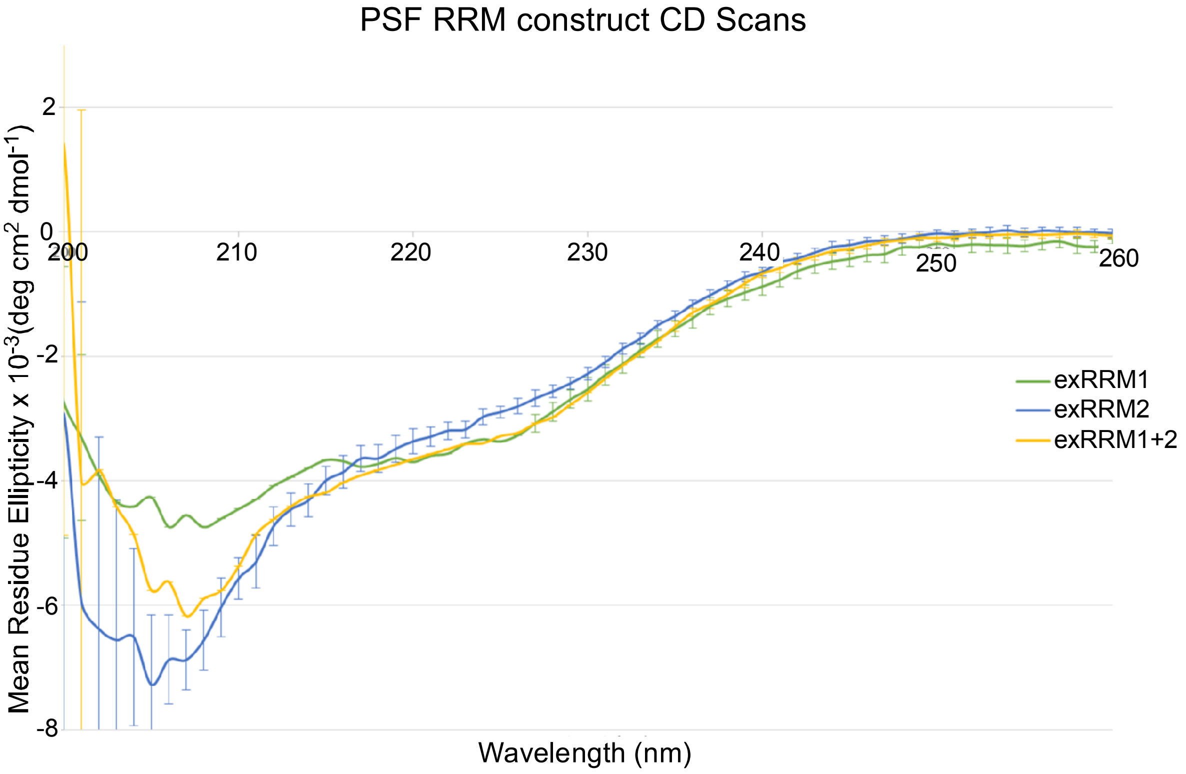

Figure S4: Circular dichroism of exRRM1, exRRM2 and exRRMs. Mean reside ellipticity for exRRM1, exRRM2, and exRRMs versus wavelength (200-300nM) is shown. Data were recorded at 25 °C using an Aviv Biomedical model 410 circular dichroism spectrometer. The protein concentration was 25 μM in all experiments, and the buffer conditions were 50 mM phosphate (pH 7.5), 150 mM NaCl for all samples. Spectra shown are the average of 3 scans. Error bars indicate standard deviation.

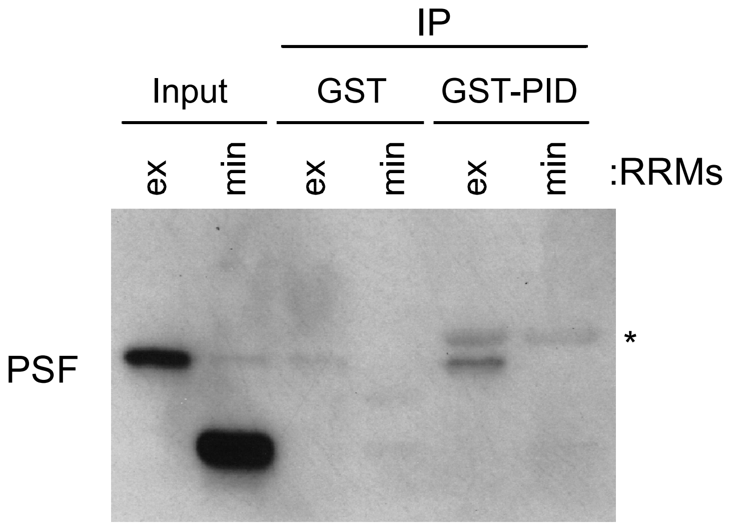

Figure S5: GST-TRAP150 PID does not pulldown HisFLAG-PSF minRRMs. Pulldown assay showing no interaction between the TRAP150 PID and PSF minRRMs despite interaction of larger exRRMs construct. Pulldowns were done as described in Materials and Methods.
